# Inhibitory Actions of Anti-Müllerian Hormone (AMH) on Ovarian Primordial Follicle Assembly

**DOI:** 10.1371/journal.pone.0020087

**Published:** 2011-05-27

**Authors:** Eric E. Nilsson, Ryan Schindler, Marina I. Savenkova, Michael K. Skinner

**Affiliations:** Center for Reproductive Biology, School of Biological Sciences, Washington State University, Pullman, Washington, United States of America; State Key Laboratory of Reproductive Biology, Institute of Zoology, Chinese Academy of Sciences, China

## Abstract

The current study was designed to investigate the actions of Anti-Müllerian Hormone (AMH) on primordial follicle assembly. Ovarian primordial follicles develop from the breakdown of oocyte nests during fetal development for the human and immediately after birth in rodents. AMH was found to inhibit primordial follicle assembly and decrease the initial primordial follicle pool size in a rat ovarian organ culture. The AMH expression was found to be primarily in the stromal tissue of the ovaries at this period of development, suggesting a stromal-epithelial cell interaction for primordial follicle assembly. AMH was found to promote alterations in the ovarian transcriptome during primordial follicle assembly with over 200 genes with altered expression. A gene network was identified suggesting a potential central role for the Fgf2/Nudt6 antisense transcript in the follicle assembly process. A number of signal transduction pathways are regulated by AMH actions on the ovarian transcriptome, in particular the transforming growth factor – beta (TGFß) signaling process. AMH is the first hormone/protein shown to have an inhibitory action on primordial follicle assembly. Due to the critical role of the primordial follicle pool size for female reproduction, elucidation of factors, such as AMH, that regulate the assembly process will provide insights into potential therapeutics to manipulate the pool size and female reproduction.

## Introduction

The functional unit within mammalian ovaries is the ovarian follicle. Each follicle has one oocyte that is surrounded by granulosa cells and theca cells [1], [2]. When ovarian follicles are first formed, they are formed as primordial follicles. The number of follicles in the primordial follicle pool is an important determinant of the reproductive lifespan of a female. Primordial follicles have an oocyte arrested in the diplotene stage of prophase I of meiosis, surrounded by flattened pre-granulosa cells [1], [2]. These primordial follicles may stay in their arrested state for months, or even years in long-lived mammals, before undergoing the primordial to primary follicle transition. Once having undergone follicle transition, the follicles will either grow and eventually ovulate, or will undergo apoptosis and follicular atresia [1], [3]. Once the pool of primordial follicles is depleted, reproduction ceases and women undergo menopause [4], [5], [6], [7].

The formation of primordial follicles is termed follicle assembly. In embryonic ovaries oogonia proliferate mitotically to form nests of germ cells that are connected by cytoplasmic bridges [8], [9], [10]. These germ cell nests become surrounded by epithelial pre-granulosa cells and have been called ovigerous cords [1], [11], [12]. The oogonia in germ cell nests enter meiosis to become oocytes and arrest at diplotene of prophase I [13], [14]. During follicle assembly the germ cell nests break down, and in mice between 1/3 and 2/3 of the oocytes are lost through apoptosis. Pre-granulosa cells invade to surround individual oocytes and so form primordial follicles [15], [16], [17]. In rats follicle assembly occurs starting on the day of birth and is mostly complete by 5 days of age, although un-assembled oocytes are sometimes detected at 10 days. In humans and cattle follicle assembly occurs in mid-gestation [1], [18], [19], [20], [21], [22].

There are some extra-cellular signaling molecules that are known to regulate follicle assembly. Progesterone and estrogen inhibit follicle assembly [23]. It is thought that changes in the levels of these hormones in the developing ovary help regulate the timing of the assembly process [23], [24], [25], [26], [27]. Tumor necrosis factor alpha (TNFα) promotes the oocyte apoptosis that is a part of follicle assembly [25], [28]. Activin is a member of the transforming growth factor beta family of signaling molecules. Treatment of mice with Activin-A at the time of follicle assembly results in more primordial follicles being formed [29]. It has also been shown that Notch, the receptor for the growth factor Jagged, is present in oocytes during follicle assembly, and that interfering with Jagged-Notch signaling inhibits the formation of primordial follicles [17], [30], [31]. In the current study, we investigate the role of Anti-Müllerian hormone (AMH), also referred to as Müllerian Inhibitory Substance (MIS), on the follicle assembly process.

Anti-Müllerian hormone is a member of the transforming growth factor beta family of extracellular signaling ligands. AMH binds to dimeric receptors comprised of the AMH-specific type 2 receptor (AMHR2), and one of three type 1 receptors: BMPR1a (Alk3), BMPR1b (Alk6) or ACVR1 (Alk2) [32], [33]. AMH was characterized for its role in causing regression of the Müllerian ducts during normal sexual development in the male fetus [34], [35]. More recently it has been found that *Amh* is expressed in ovaries from the granulosa cells of secondary to early antral follicles. Previous studies have shown AMH acts to inhibit primordial to primary follicle transition, and so maintains primordial follicles in their arrested state [36], [37], [38], [39].

Earlier micro-array experiments in our laboratory showed that *Amh* mRNA expression levels were altered in the ovaries of new-born rats, in which oocytes were present in nests, when compared to four-day old ovaries, in which oocytes were mostly assembled into primordial follicles [40]. In ovaries of this age there are no secondary or later stage follicles present from which AMH may be produced. This opens questions about the source of AMH in these neonatal ovaries, and what the role of AMH might be during this time of follicle assembly. In the current study rat ovary organ culture experiments are used to characterize the actions of AMH during the time of follicle assembly, when the pool of primordial follicles is formed. The hypothesis tested is that AMH regulates the rate of assembly of oocytes into primordial follicles. Immuno-histochemical experiments are performed to determine the localization of AMH protein expression. Microarray experiments are also performed to determine how gene expression changes in neonatal ovaries after AMH treatment. Observations elucidate the regulation of primordial follicle assembly, which is the process that forms the pool of primordial follicles, and so ultimately affects the fertile lifespan of women and all other female mammals.

## Results

**Figure 1:**
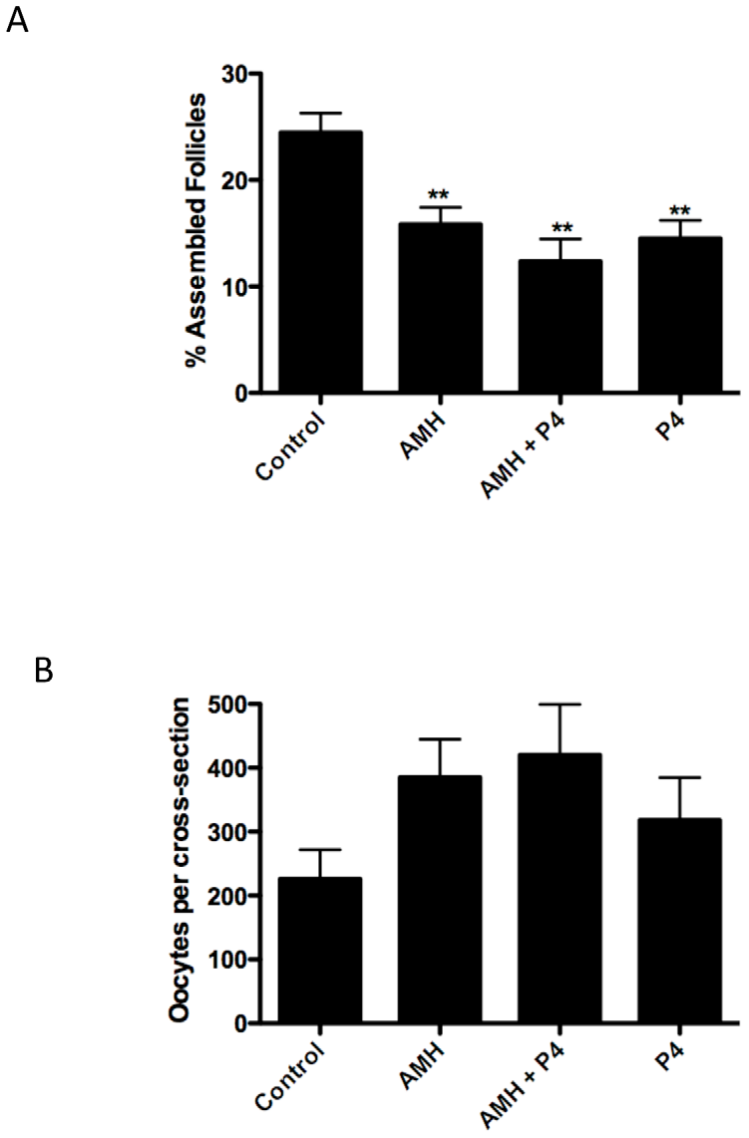
Primordial follicle assembly after 2 days of treatment (A) represented as % Assembled Follicles after AMH, progesterone (P_4_) and AMH combined with P_4_ treatments. Oocyte number per cross section (B) after the same treatments. The Mean ±SEM from a minimum of five different experiments performed in replicate with statistical significance indicated as (**) (P<0.01).

Whole ovaries from zero-day old rat pups were cultured for either two or ten days in the presence or absence of AMH. Zero-day old ovaries after two days of culture have partially completed the follicle assembly process [23]. Treatments that affect the rate of follicle assembly may be readily evaluated at this time. At the end of culture, the ovaries were fixed, sectioned and stained, and then examined microscopically. The number of oocytes that had been assembled into follicles was determined, as was the number of oocytes that remained un-assembled in nests. Ovaries cultured for two days were treated with AMH (50 ng/mL), progesterone (P_4_)(10^−6^ M), or the combination of AMH and P_4_. Progesterone has previously been shown to slow the rate of follicle assembly [23], and was used as a positive control in these experiments. Treatment with AMH resulted in a significant (p<0.01) decrease in the percentage of assembled follicles compared to untreated controls (Figure 1A). Treatment with P_4_, as expected, resulted in a significant (p<0.01) decrease in assembled follicles. The combination of AMH and P_4_ also resulted in a significant (p<0.001) decrease in assembled follicles compared to controls (Figure 1A). A corresponding increase in oocyte number compared to controls was observed in the AMH treated ovary cultures (Figure 1B), suggesting the apoptosis of oocytes was inhibited resulting in an increase in oocyte number. AMH-induced alterations in oocyte apoptosis may be an aspect of the mechanism in the inhibition of follicle assembly. These results indicate that AMH can slow the rate of follicle assembly.

**Figure 2:**
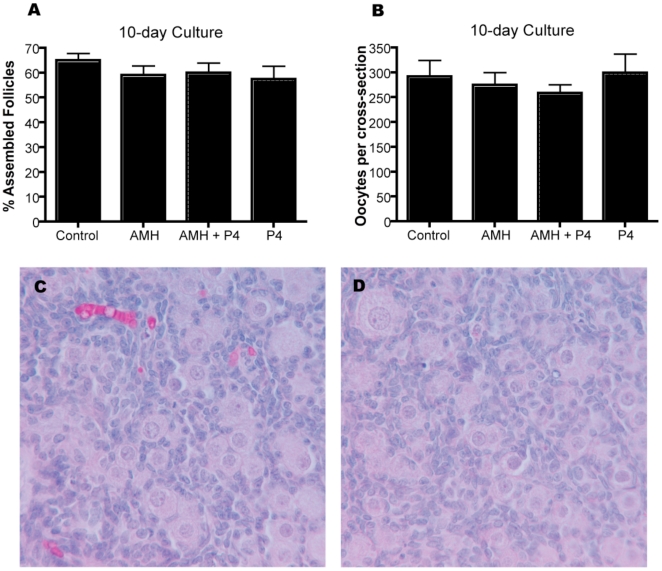
Primordial follicle pool size and percentage of assembled follicles as determined by oocyte counts per cross section after 10 days of treatment with AMH, progesterone (P_4_) and AMH combined with P_4_. The mean ±SEM for a minimum of three experiments in replicate performed. (A) Percent assembled follicles. (B) Total oocytes per cross-section. (C) Representative untreated control ovary section, H&E stained. (D) Representative AMH treated ovary section, H&E stained.

Rat ovaries were cultured for ten days to determine if AMH treatment could have an effect on the size of the assembled primordial follicle pool. Ovaries cultured for ten days, similarly to those treated for two days, were treated with AMH (50 ng/mL), P_4_ (10^−6^ M), or the combination of AMH and P_4_. Results indicate none of the treatments affected oocyte number after 10 days of treatment (Figure 2B). Therefore, the alterations in primordial follicle assembly regarding primordial follicle pool size was corrected after 10 days of culture. Similarly, there was no difference in the percentage of assembled follicles between treatments after 10 days (Figure 2A). Ovaries cultured for 10 days appeared healthy with minimal necrosis of follicular cells. Un-assembled oocytes were present in small nests of 2–3 oocytes (Figure 2C and 2D).

**Figure 3:**
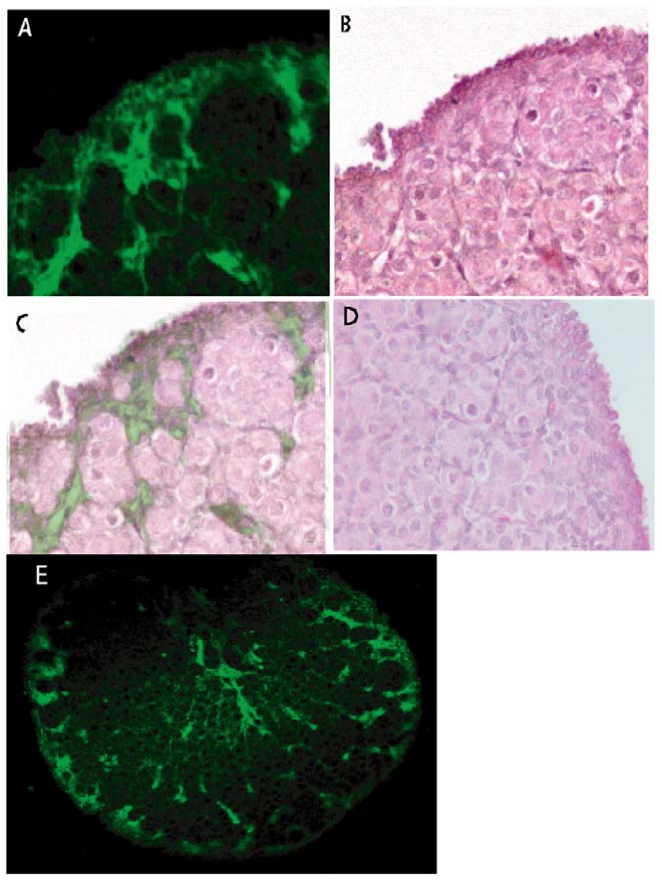
Ovarian morphology and immunohistochemical localization of AMH. (A) Fluorescent immunohistochemical localization of AMH protein (green) in cortex of 3-day old rat ovary. (B) Hematoxylin/eosin (H and E) stain of the same ovarian section as was used for immunohistochemistry. (C) Overlay of immunohistochemical localization of AMH (green) onto H and E stained section (i.e. A onto B). AMH is present in the stromal cells around groups of forming and newly formed primordial follicles. (D) An additional H and E stained ovarian cross-section from the same ovary, with more clear morphological detail than was possible with the re-stained section in B. (E) Immunohistochemical localization of AMH across the entire ovarian cross-section, showing AMH to be present near the ovarian cortex and in stromal bands in tzhe medulla.

Immunohistochemistry was performed to determine where AMH protein was localized in neonatal rat ovaries (Figure 3). Confocal fluorescent microscopy showed that AMH was localized to stromal cells surrounding oocyte nests and primordial follicles (Figure 3A and 3C). AMH protein did not appear to be present in oocytes. Examining the pattern of AMH expression across an ovary cross-section reveals that there is a region of high expression levels near the ovarian cortex around forming follicles, and also expression in selected bands of stromal tissue in the interior of the ovary (Figure 3E). The stromal localization of AMH at this period of ovarian development suggests an important stromal-epithelial interaction in follicle assembly.

**Figure 4:**
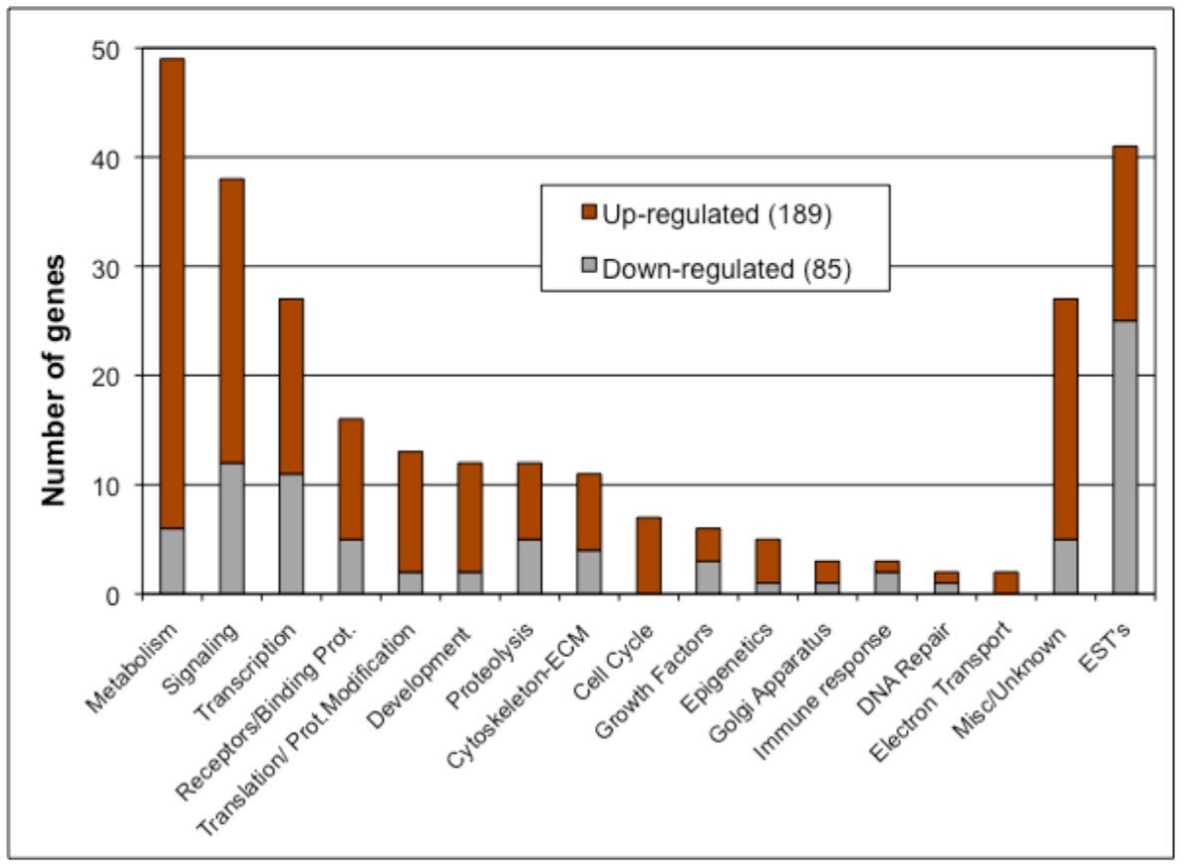
Number of genes differentially expressed in rat P0-ovary upon AMH treatment and their distribution among main functional categories. Dark-grey - up-regulated (189 genes) and light-grey - down-regulated genes (85 genes).

**Figure 5:**
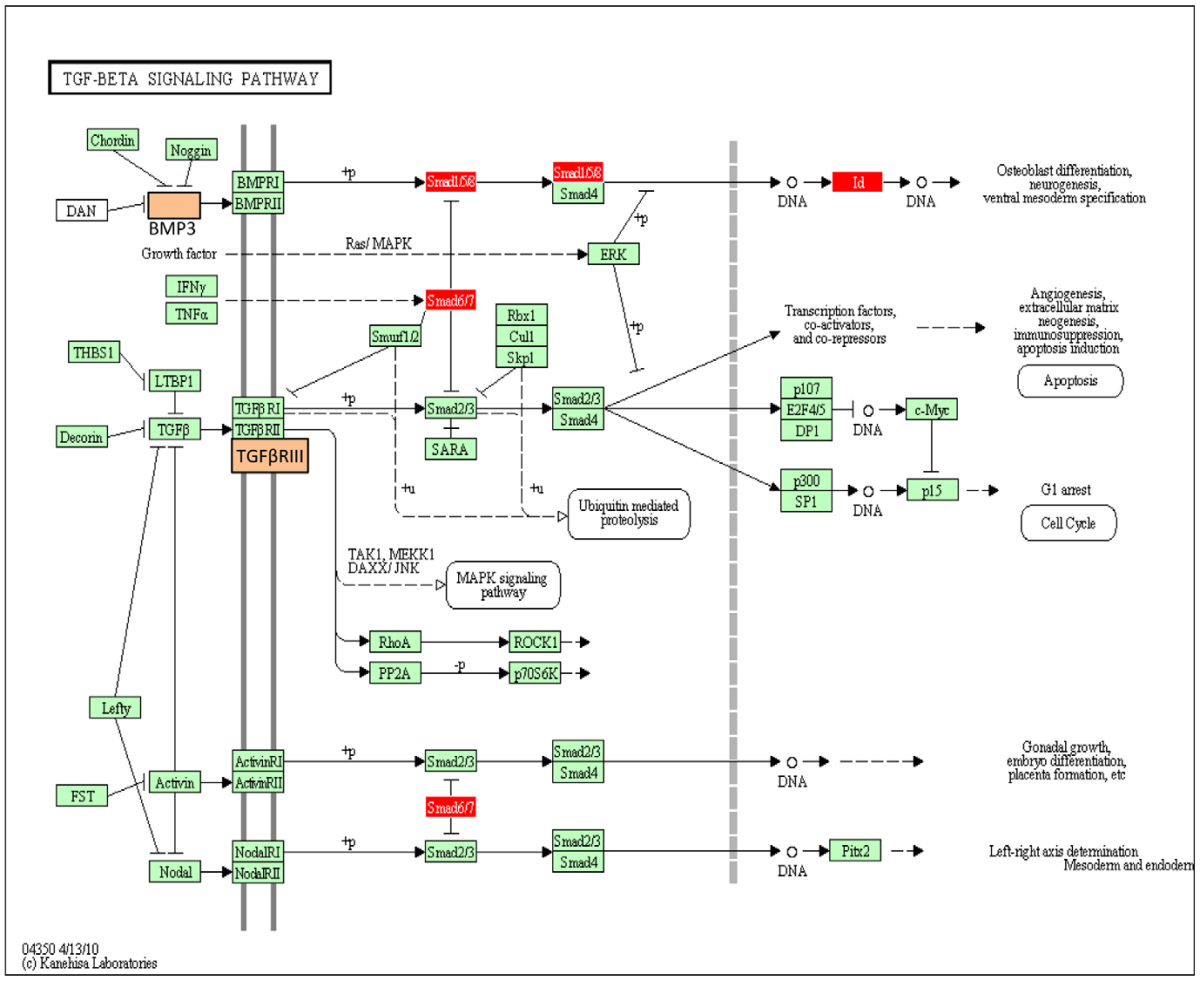
TGF-beta Signaling Pathway (KEGG) showing rat P0-Ovary genes affected by AMH treatment. Red boxes – up-regulated, green and white boxes –not affected genes.

**Figure 6:**
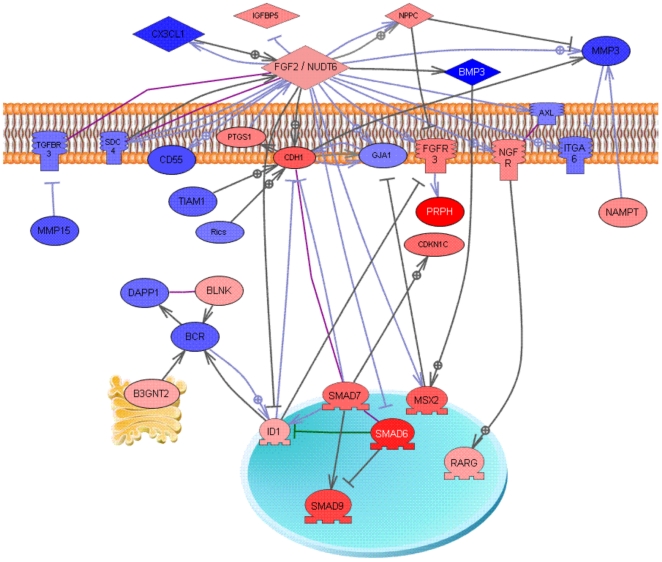
Gene network with direct connection scheme for AMH treated P0-ovary differentially expressed genes. Only 31 genes directly connected from the list of 274 are shown according to their location in the cell (on membrane, in Golgi apparatus, nucleus, cytoplasm or outside the cell), the rest of genes are not connected and not shown. Node shapes code: oval and circle – protein; diamond – ligand; circle/oval on tripod platform – transcription factor; ice cream cone – receptor. Red color represents up-regulated genes, blue color – down-regulated genes; arrows with plus sign show positive regulation/activation, arrows with minus sign – negative regulation/inhibition; grey arrows represent regulation, lilac - expression, and purple – binding.

RNA was collected from rat ovaries that had been placed into culture at zero days of age and cultured with or without AMH treatment for one day. A 24-hour treatment was not found to effect any morphology or follicle development (data not shown), such that any effects in the transcriptome are not due to differential morphogenesis of the ovary. The RNA from each treatment group was subjected to microarray analysis using Affymetrix Rat Gene 1.0 ST chips. The microarray data for the different arrays were found to match very well after processing of the data (Figure S1). Compared to controls, AMH treated ovary RNA showed changes in the expression of 285 probe sets, mapping to 274 different genes (Figure S2). This list of altered genes (Table S1) was analyzed using several different methods to gain insights into the functional processes impacted by AMH signaling. The genes were categorized into functional categories that demonstrated metabolism, signaling and transcription functions were the most highly represented, Figure 4 and Table S2. The majority of genes were up-regulated (189) with a smaller number down-regulated (85), Figure 4. The list of differentially expressed genes was also analyzed to see what well-characterized cellular signaling pathways these genes fell into using KEGG (Kyoto Encyclopedia of Genes and Genomes) pathway analysis. The altered genes were found to associate with several signaling pathways (Table 1). The most highly represented pathway with the highest impact was the TGFß signaling pathway, Figure 5. The expression of a number of critical TGFß signal transduction genes were modified. Other signaling pathways effected were calcium signaling and cell adhesion molecule signaling among the KEGG pathways impacted by these genes (Table 1). An automated analysis of the 274 differentially expressed genes (Pathway Studio, Ariadne, Inc.) revealed that a subset of these genes were associated with a gene network. A sub-network formed from these related and connected genes is presented in Figure 6 that demonstrates a potential central role for the *Nudt6* antisense gene for the fibroblast growth factor-2 gene (*Fgf2*). The cellular processes that this signaling event regulates had a high degree of connectivity with the AMH altered genes (Table S2). This gene network will be important to consider in regulating primordial follicle assembly. A more global view of signaling events influenced by AMH are presented in Figure S3. A number of different signaling pathways appear to be altered by AMH actions on primordial follicle assembly, Table 1 and Figure S3.

**Table 1:** Top KEGG signaling pathways enriched with genes differentially expressed in P0 ovary upon AMH treatment (274 genes).

Pathway Name	Impact Factor	Differentially Regulated Genes in Pathway	Total Genes in Pathway
TGF-beta signaling pathway	9.35	5	81
Calcium signaling pathway	5.87	4	189
General signaling pathways (Pathways in cancer)	5.19	6	321
Melanogenesis	4.61	3	96
Cell adhesion molecules (CAMs)	4.29	5	151
Regulation of actin cytoskeleton	4.02	4	204
Cell cycle	3.10	3	117
Neuroactive ligand-receptor interaction	2.48	5	259
ECM-receptor interaction	2.42	3	74
Axon guidance	2.27	3	124
Endocytosis	*	4	226
Glycerophospholipid metabolism	*	3	76
Arrhythmogenic right ventricular cardiomyopathy (ARVC)	*	3	71
Malaria	*	3	56

Asterisks (*) indicates a pathway not in ONTO-tools database (see Methods), so, impact factors for them have not been calculated.

## Discussion

Previous investigations demonstrated that AMH was expressed in the ovaries of new-born rats and the level of *Amh* mRNA expression was different in zero-day old ovaries containing oocyte nests when compared to four-day old ovaries having mostly assembled primordial follicles [40]. The current studies were designated to determine what role anti-Müllerian hormone (AMH) has in regulating the formation of primordial follicles. Organ culture experiments were performed in which neonatal rat ovaries were treated with AMH during the time of follicle assembly. Both zero-day old rat ovaries cultured for two days and *in vivo* ovaries from two-day-old rats have partially completed the follicle assembly process [1], [2], [3], [23]. Experimental treatments either *in vitro* or *in vivo* that affect the rate of follicle assembly may be readily evaluated at this time point. *In vivo* the follicle assembly process is essentially completed by 4–5 days of age. However, when zero-day-old rat ovaries are cultured for 4 to 6 days, a larger portion of oocytes remain un-assembled in nests compared to what is seen *in vivo* [23]. Even after ten days of culture, small oocyte nests are retained, both in the current study and in previous research [41]. So evaluations of follicle pool size are performed after ten days of culture, when oocyte loss is expected to have stabilized.

AMH treatment for two days inhibited primordial follicle assembly (Figure 1). The affect of AMH was similar to that of progesterone, a previously identified inhibitor of follicle assembly in rats and cattle [23], [24], [26]. A corresponding increase in oocyte number was associated with the inhibition of follicle assembly. The majority of oocytes undergo apoptosis during follicle assembly, so AMH appears to have reduced apoptosis to inhibit assembly. These results indicate that AMH acts to inhibit the rate of primordial follicle formation. This is one of the first growth factors shown to inhibit primordial follicle assembly. After ten days of culture only small oocyte nests are present [41], and no difference in the primordial follicle pool was observed. The cultured ovaries appeared morphologically healthy. Therefore, alterations during primordial follicle assembly were corrected after ten days of ovarian culture. The actions of AMH appear to be focused on the initial stages of primordial follicle assembly.

Immunohistochemical studies showed that AMH protein was localized to the ovarian stromal cells surrounding germ cell nests and newly formed primordial follicles (Figure 3). These stromal cells appear to be the source of the AMH that acts to inhibit follicle assembly. This is different than what is seen in the ovaries of older animals. In ovaries from adult animals AMH acts to inhibit the transition of arrested primordial follicles to developing primary follicles [42]. In these adult ovaries AMH is produced from the granulosa cells of developing follicles from the late secondary to early antral stages [36], [37], [38], [39]. Granulosa cells are an epithelial cell type, and so are distinct from the stromal cells where AMH is localized in the younger animals of the current study. Observations suggest an important stromal-epithelial cell interaction during primordial follicle assembly. Mutant mice mull for the AMH gene can still successfully form primordial follicles [38], so AMH signaling may modulate the timing of initiation of follicle assembly, and likely acts in concert with other regulatory growth factors. Even though the sites of AMH localization are different, it is interesting that the role of AMH in both neonatal and adult ovaries is to inhibit the progression of primordial follicle development. A recent study [43] reported AMH expression in developing primary follicles of 4-day old rat ovaries, rather than in surrounding stroma. In the 3-day old ovaries evaluated in the current study, follicle assembly was not complete, and no developing primary follicles were apparent. This raises the possibility that there is a switch in AMH expression from stromal to epithelial cell types at the completion of follicle assembly.

The mRNA from 0-day old ovaries treated with AMH was compared with that of untreated controls using a microarray analysis. It was found that transcripts from 274 genes were differentially expressed in AMH-treated ovaries. Genes from this list are known to be involved in processes of cell signaling, differentiation, proliferation and apoptosis, as might be expected in ovaries undergoing assembly of primordial follicles. AMH is a member of the TGFß family of growth factors. Several of the differentially expressed genes known to be involved in TGFß family signaling pathways including *Smad6*, *Smad7*, *Smad9*, *Tgfbr3* (betaglycan) and the additional signaling ligand gene *Bmp3* (bone morphogenetic protein 3) were found to be regulated by AMH (Figure 5). This further indicates that TGFß family signaling is important to the follicle assembly process. BMP growth factors have previously been shown to be expressed in adult rat ovaries in theca cells of antral follicles [44] and influence primordial follicle development [45]. The TGFß growth factor can inhibit cell proliferation, inhibit apoptosis and promote cell survival [46]. TGFß can promote the cell cycle inhibitor p27 to arrest the cell cycle G1 phase [47], directly inhibit the apoptosis signaling pathway [47], and promote extracellular matrix production and cell survival [48]. The actions of AMH observed suggest a similar signal transduction pathway, Figure 5, during primordial follicle assembly blocks oocyte apoptosis to inhibit follicle assembly. Observations suggest this may be the primary mechanism for AMH actions.

A number of genes for additional growth factors and extracellular signaling ligands were also differentially expressed in AMH-treated ovaries (Table S1). *Cx3cl1* has been shown to have a role in adult ovary progesterone synthesis [49], but its role in primordial follicle development has not been investigated. Similarly, *Rspo1* is known to have a role in embryonic ovarian differentiation [50], [51], [52], but its role in later stages of ovarian development have not been examined. Other signaling ligands such as *Creg1*, *Ecop* and *Megf6* were not previously known to be expressed in ovaries. Further research is needed to determine if any of these genes are involved in primordial follicle assembly. As previously suggested [1], a network of growth factor mediated interactions will likely be required to regulate primordial follicle development.

A gene network was identified of the most interactive and connected genes within the AMH differentially regulated genes, Figure 6. The genes *Bmp3* and the *Smad* transcription factors were present, further confirming the role of this pathway in the actions of AMH on follicle assembly. A central regulatory factor in the gene network identified is the *Nudt6* antisense transcript for the fibroblast growth factor 2 gene (*Fgf2*) expression product. The *Nudt6* is an embedded reverse transcript within the *Fgf2* gene [53], [54] that has the capacity to inhibit the expression and action of FGF2 [55], [56]. This FGF2/NUDT6 relationship indicates a critical regulatory role in the gene network identified, Figure 6, to influence signal transduction processes and transcriptional regulation. The observations of the current study suggest these genes and the network identified are potential candidates involved in the regulation of primordial follicle assembly. Future research on the role of these genes and this network is anticipated to help elucidate the molecular regulation of primordial follicle assembly.

A recent study demonstrated that a bionetwork of interacting genes was important for regulating the transition of arrested primordial follicles into developing primary follicles [57]. An important finding from that study was that several growth factors that affected follicle transition also impacted many of the same signaling pathways, but that each different growth factor affected different genes within those pathways. In the current study it may be that AMH affects expression of a different set of genes than other factors impacting follicle assembly, but that other regulatory factors effect similar pathways. Studies are in progress to test this hypothesis with a bionetwork analysis of primordial follicle assembly.

The primordial follicle pool size and development helps determine the reproductive capacity of most mammalian species. Abnormal primordial follicle development is associated with premature ovarian failure and menopausal conditions [1]. The factors that control the assembly of primordial follicles have a role in establishing the follicle pool size and so indirectly the reproductive capacity of a female. The current study has identified AMH as an important regulator of primordial follicle assembly that appears to mediate a stromal-epithelial interaction in the developing ovary to inhibit follicle assembly. Perhaps AMH from stromal cells has an important role in maintaining the oocyte nests to prevent premature follicle assembly. The balance of inhibitory signals such as AMH and progesterone with growth factors that promote follicle assembly such as TNFα, activin and jagged will determine whether follicle assembly will proceed. In the current study a gene network potentially involved in primordial follicle assembly was identified and may be used to design therapeutic agents to manipulate the primordial follicle pool size. Potential additional regulatory agents for follicle assembly were also identified.

## Methods

### Treatments and ovary culture

Zero-day old female Sprague-Dawley rats (Harlan Laboratories, Inc., USA) were euthanized according to Washington State University IACUC approved protocols and their ovaries removed and cultured whole as described previously [58]. All procedures were approved by the Washington State University Animal Use and Care Committee (IACUC approval # 02568-018). Zero-day old rat ovaries contain almost exclusively nests of un-assembled oocytes. Whole ovaries were cultured on floating filters (0.4 µm Millicell-CM, Millipore, Bedford, MD, USA) in 0.5 ml Dulbecco's modified Eagle's medium (DMEM)-Ham's F-12 medium (1∶1, vol/vol) containing 0.1% BSA (Sigma), 0.1% Albumax (Gibco BRL, Gaithersburg, MD, USA), 27.5 µg/ml transferrin, and 0.05 mg/ml L-ascorbic acid (Sigma) in a four-well culture plate (Nunc plate, Applied Scientific, South San Francisco, CA, USA) for two or ten days. Previous studies have shown that zero-day-old ovaries cultured for ten days have good cell viability [41]. The medium was supplemented with penicillin and streptomycin to prevent bacterial contamination. Ovaries were randomly assigned to treatment groups, with 1–3 ovaries per floating filter per well. Wells were treated every two days with recombinant AMH (human Anti-Müllerian Hormone)(50 ng/mL, R&D Systems Inc., USA) and/or progesterone (P_4_) (Sigma) at 10^−6^ M. Pilot studies in our laboratory indicated that 50 ng/mL AMH had a maximal effect on primordial to primary follicle transition in cultured neonatal rat ovaries (data not shown). After culture, ovaries were fixed in Bouin's fixative (Sigma) for two hours. Ovaries were then embedded in paraffin, sectioned at 3 µm and stained with hematoxylin/eosin for use in morphological analysis.

### Morphological Analysis

For each ovary the number of oocytes at each developmental stage was counted and the counts for each stage were averaged across the two consecutive histological sections that had the largest ovarian cross section. The oocyte nucleus had to be visible for an oocyte to be counted. Normally, between 100 and 300 follicles were present in each cross-section. Oocytes were classified as being not yet assembled into follicles (i.e. the oocyte was part of an unassembled oocyte nest), as primordial (stage 0) or as one of the developing pre-antral stages (stages 1–4) as described previously [23], [59]. Unassembled oocytes are recognized from their being grouped together without any intervening stromal cells. Primordial follicles consist of an oocyte partially or completely surrounded by a single layer of squamous pre-granulosa cells. Follicles with only one cuboidal granulosa cell visible, the rest being squamous, were also classified as primordial [60], [61], [62], [63]. Developing (stages 1–4) follicles contain successively more cuboidal granulosa cells in layers around the oocyte [59], [64].

### Immunohistochemistry

Ovary sections from 3-day old un-cultured rat ovaries were immunostained as described previously [65], for the presence of AMH using an anti-AMH primary antibody (anti-AMH goat IgG; R&D Systems, USA). Briefly, 3 µm sections were de-paraffinized, rehydrated through a graded ethanol series, boiled in 10 mM sodium citrate buffer, washed with 0.1%Triton-X solution, and then blocked with 10% goat serum (normal goat serum; Vector Laboratories Inc., Burlingame, CA, USA) for 20 min prior to incubation with 5 µg/ml primary antibody for 12 h. The sections were then washed with PBS and incubated with 1∶3000 diluted conjugated secondary antibodies for 45 min (Alexafluor donkey anti-goat IgG and Alexafluor donkey anti-mouse IgG; Invitrogen), and then again washed several times before mounting with cover slips. Images were captured using a con-focal fluorescent microscope (Zeiss 510). Positive control sections of testes showed the expected staining of Sertoli cells (data not shown), confirming the specificity of the anti-AMH antibody. Negative control experiments were performed using non-specific primary antibody at 5 µg/ml (goat IgG; R&D Systems).

### RNA Collection

For ovary culture experiments in which ovarian RNA was collected, 2–3 ovaries per well were cultured for one day either untreated (controls) or were treated with AMH (human Anti-Müllerian Hormone) (50 ng/mL, R&D Systems Inc., USA). After one day of culture there are no morphological differences between control and growth factor-treated ovaries (data not shown), so measurements of whole-ovary gene expression will reflect differences in RNA transcription, rather than differing proportions of cell types due to differential cell proliferation between treatments.

RNA was isolated from whole rat ovaries after homogenization in one mL Trizol™ reagent (Sigma-Aldrich, USA), according to manufacturer's instructions. 2–3 ovaries from the same culture well (from different rat pups out of the same litter) and receiving the same treatment were pooled and homogenized together. Homogenized samples were stored at −70C until the time of RNA isolation.

### Microarray transcriptome analysis

The mRNA processing and hybridization were performed at the Genomics Core Laboratory, Center for Reproductive Biology, Washington State University, Pullman, WA using standard Affymetrix reagents and protocol. Briefly, mRNA was transcribed into cDNA with random primers, from the later, cRNA was transcribed, and from that, single-stranded sense DNA was synthesized which was fragmented and labeled with biotin. Biotin-labeled fragmented ssDNA was then hybridized to the Rat Gene 1.0 ST microarrays containing more than 27,000 transcripts (Affymetrix, Santa Clara, CA, USA). Hybridized chips were scanned on Affymetrix Scanner 3000. CEL files containing raw data were then pre-processed and analyzed with Partek Genomic Suite 6.5 beta software (Partek Incorporated, St. Louis, MO) using an RMA and GC-content adjusted algorithm. The signals from an average of 28 different probes for each transcript were compared to give a single value. Lists of differentially expressed genes for each treatment were generated using following cut off criteria: signal ratio Control/Treatment >1.20 change, mean difference for un-logged signals between control and treatment >10, t-test p-values<0.05, and correcting for organ culture date batch effects.

CEL files (MIAME compliant raw data) from this study have been deposited with the NCBI gene expression and hybridization array data repository (GEO, http://www.ncbi.nlm.nih.gov/geo, #GSE 25629) and can be also accessed through www.skinner.wsu.edu. For gene annotation, Affymetrix annotation file RaGene1_0stv1.na31.rn4.transcript.csv was used unless otherwise specified. Generation of affected KEGG pathways (Kyoto Encyclopedia for Genes and Genome, Kyoto University, Japan) used Pathway-Express, a web-based tool freely available as part of the Onto-Tools (http://vortex.cs.wayne.edu) [66].

Previous studies have demonstrated that microarray data are validated with quantitative PCR data [40], [67]. Due to the presence of an average of 28 different oligonucleotide probes for each specific gene being used on the microarray versus only a single primer set for a gene in a quantitative PCR, the microarray is more effective at eliminating false positive or negative data and provides a more robust quantification of changes in gene expression.

### Statistical Analysis

Treatment groups are compared using analysis of variance (ANOVA) followed by Dunnet's post-hoc tests where appropriate. Groups were considered statistically significant with P≤0.05. All statistics were calculated using Graph Pad Prism version 5.0b for Macintosh, Graph Pad Software, San Diego, CA, USA.

## Supporting Information

Figure S1 **Sample histograms and box plots for microarray raw data (A) and pre-processed signal values, using RMA, GC-content adjusted algorithm (B) for control (blue) and 3 AMH treated (red) chips.**
(PDF)

Figure S2 **Heatmap for AMH-treated P0-ovary 274 differentially expressed genes obtained with Partek GS 6.5 software.** Means for control and AMH samples are shown: highly expressed genes colored in red, low expressed in green, medium expressed in yellow.
(PDF)

Figure S3 **General Signaling Pathways showing rat P0-ovary genes affected by AMH treatment: red boxes – up regulated, blue – down-regulated, green and white boxes – not affected genes.**
(PDF)

Table S1 **Genes expressed differen6ally in P0 Ovaries upon an6-Mullerian (AMH) hormone treatment.**
(PDF)

Table S2 **Top cell processes for 274 differentially expressed genes in AMH-treated P0-ovary.** Cell processes with highest local connectivity values (number of related literature references) were extracted from shortest connection subnetwork obtained with Pathway Studio 7.0 software (Ariadne Genomics, Inc., Rockville, MD).
(PDF)
