# Supplementary figures and images for: Inhibitory Actions of Anti-Müllerian Hormone (AMH) on Ovarian Primordial Follicle Assembly
Source: PLoS One. 2011 May 27;6(5):e20087. doi: 10.1371/journal.pone.0020087 (PMC3103528; doi:10.1371/journal.pone.0020087)

A

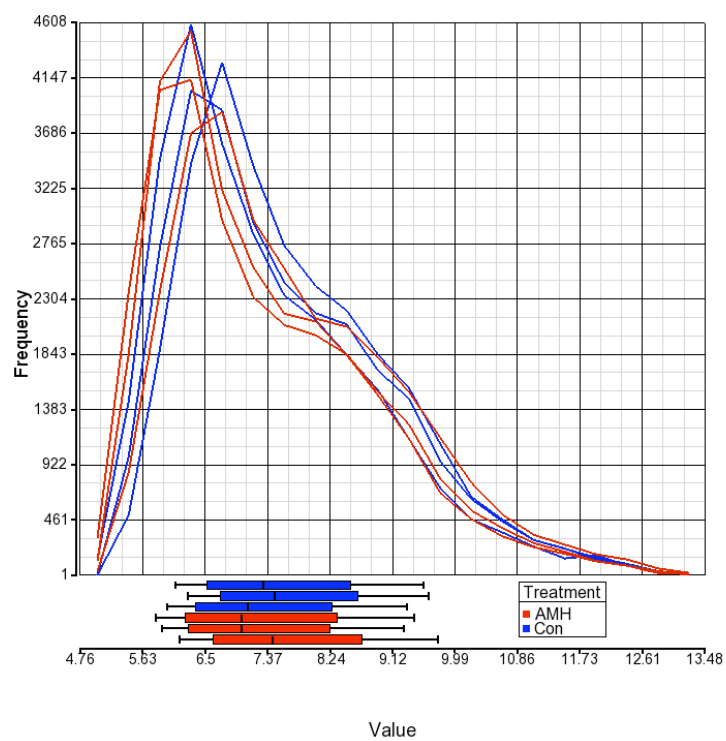

B

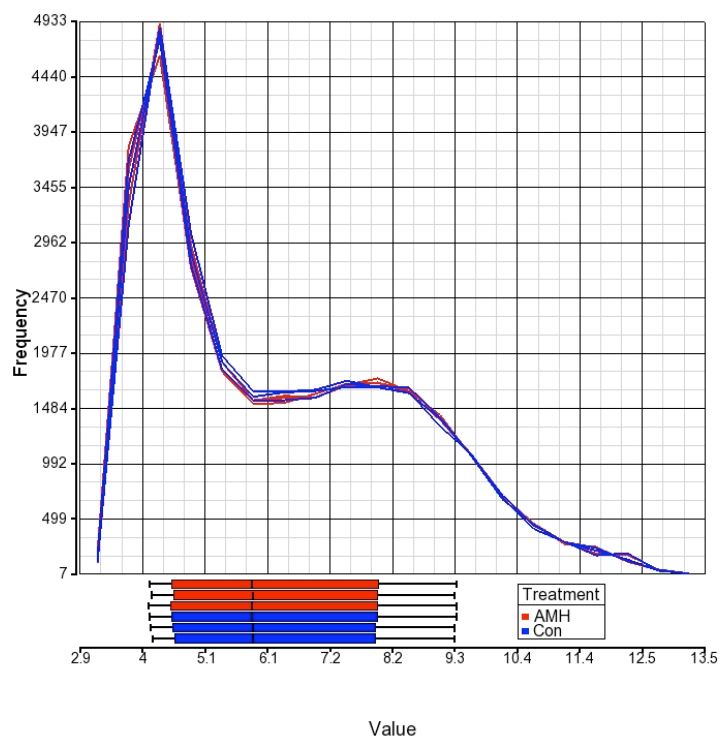

Supplemental Figure S1 (Color)

Supplement: Figure S1 — Sample histograms and box plots for microarray raw data (A) and pre-processed signal values, using RMA, GC-content adjusted algorithm (B) for control (blue) and 3 AMH treated (red) chips. (PDF) [file pone.0020087.s001.pdf]

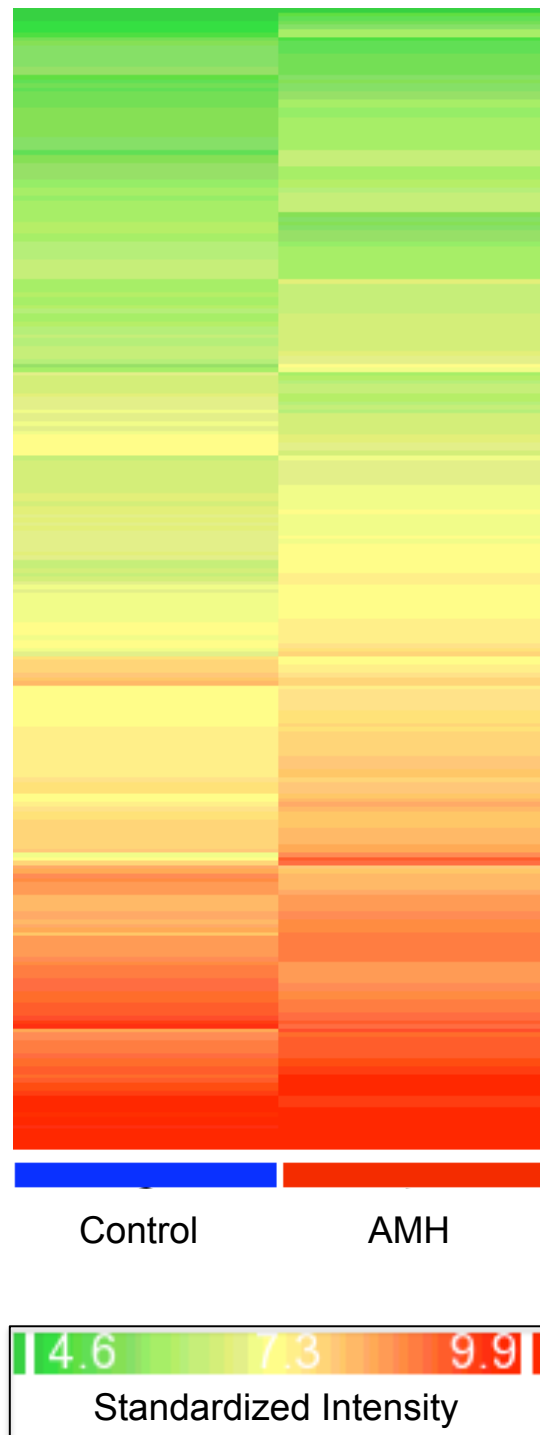

Supplemental Figure S2

Supplement: Figure S2 — Heatmap for AMH-treated P0-ovary 274 differentially expressed genes obtained with Partek GS 6.5 software. Means for control and AMH samples are shown: highly expressed genes colored in red, low expressed in green, medium expressed in yellow. (PDF) [file pone.0020087.s002.pdf]

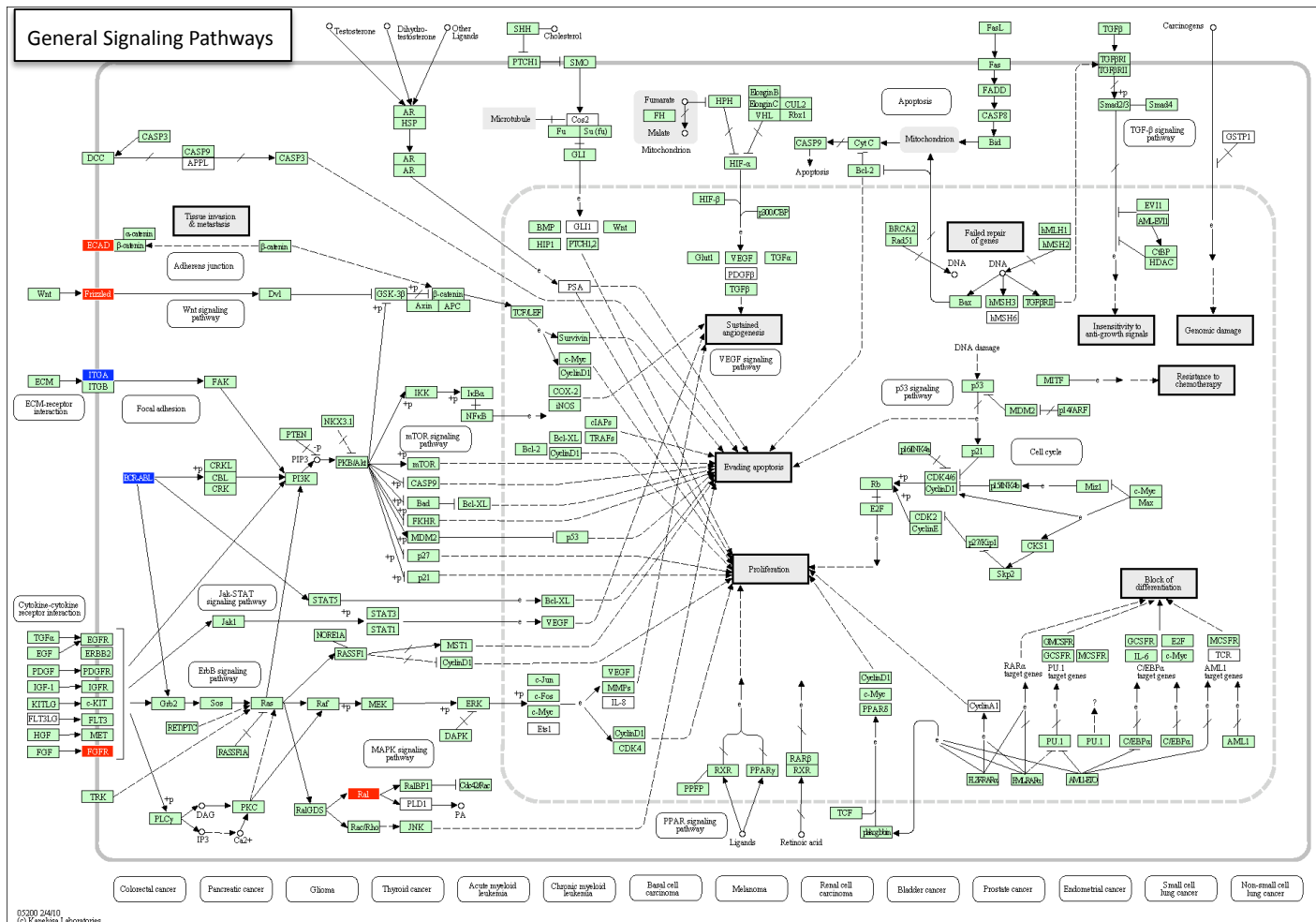

Supplemental Figure S3

Supplement: Figure S3 — General Signaling Pathways showing rat P0-ovary genes affected by AMH treatment: red boxes – up regulated, blue – down-regulated, green and white boxes – not affected genes. (PDF) [file pone.0020087.s003.pdf]
