# Supplementary material for: Inhibitory Actions of Anti-Müllerian Hormone (AMH) on Ovarian Primordial Follicle Assembly
Source: PLoS One. 2011 May 27;6(5):e20087. doi: 10.1371/journal.pone.0020087 (PMC3103528; doi:10.1371/journal.pone.0020087)
Supplement: Table S1 — Genes expressed differen6ally in P0 Ovaries upon an6-Mullerian (AMH) hormone treatment. (PDF) [file pone.0020087.s004.pdf]

Supplemental Table 1.
Genes expressed differentially in P0 Ovaries upon anti-Mullerian (AMH) hormone treatment

Gene Symbol	*GenBank, Reference Sequence*	*mean_dif (AMH-Con)*	*Ratio AMH/Con*	*Affymetrix Probe Set ID*	*Gene Title*
**Cell Cycle**					
Cdc45l	NM_001105866	1.21	21.5	10755992	CDC45 cell division cycle 45-like (S. cerevisiae)
Cdc20	NM_171993	1.25	41.8	10879257	cell division cycle 20 homolog (S. cerevisiae)
Ccng2	NM_001105725	1.48	327.3	10775761	cyclin G2
Cdkn1c	NM_001033757	1.69	120.3	10727056	cyclin-dependent kinase inhibitor 1C
Cdkl4	ENSRNOT00000061975	1.26	24.6	10888046	cyclin-dependent kinase-like 4
Hjurp	ENSRNOT00000051065	1.20	25.6	10929713	Holliday junction recognition protein
Spin2a	NM_001127657	1.28	46.5	10937455	spindlin family, member 2A
**Cytoskeleton-ECM**					
Blnk	NM_001025767	1.28	13.2	10730131	B-cell linker
Cdh1	NM_031334	1.90	84.3	10807542	cadherin 1
Celsr1	ENSRNOT00000036025	0.80	-19.2	10905988	cadherin, EGF LAG seven-pass G-type receptor 1 (flamingo homolog, Drosophila)
Chsy1	NM_001106268	1.41	153.7	10707832	chondroitin sulfate synthase 1
Dctn3	NM_001108659	1.23	28.5	10876172	dynactin 3
Kank4	NM_001107947	0.81	-13.0	10878210	KN motif and ankyrin repeat domains 4
Lce1f	NM_001109188	0.82	-17.3	10817134	late cornified envelope 1F
Prph	NM_012633	3.68	423.8	10899061	peripherin
Plod1	NM_053827	1.29	23.8	10742968	procollagen-lysine 1, 2-oxoglutarate 5-dioxygenase 1
Spock2	NM_001108533	1.26	95.0	10829965	sparc
Sdc4	NM_012649	0.80	-380.4	10851599	syndecan 4
**Development**					
Cln6	ENSRNOT00000034926	1.24	74.0	10910708	ceroid-lipofuscinosis, neuronal 6
Crispld2	NM_138518	1.27	16.3	10808377	cysteine-rich secretory protein LCCL domain containing 2
Dmc1	NM_001130567	1.25	28.0	10897666	DMC1 dosage suppressor of mck1 homolog, meiosis-specific homologous recombination (yeast)
Lrrc46	NM_001004201	1.22	29.9	10746619	leucine rich repeat containing 46
Metrnl	NM_001014104	1.50	58.4	10931247	meteorin, glial cell differentiation regulator-like
Ntn3	NM_053732	1.23	11.1	10741009	netrin 3
Prrx2	NM_001105739	1.55	14.6	10835257	paired related homeobox 2
Rai2	NM_001109316	1.21	35.5	10937834	retinoic acid induced 2
LOC691681	ENSRNOT00000052385	0.80	-102.5	10809503	similar to Discs large homolog 5 (Placenta and prostate DLG) (Discs large protein P-dlg)
Syn3	NM_017109	0.80	-10.1	10894465	synapsin III
Bco2	XM_001074312	1.31	74.8	10917231	beta-carotene oxygenase 2
Vangl2	NM_001105969	1.40	27.5	10769919	vang-like 2 (van gogh, Drosophila)
**Electron Transport**					
Cybrd1	NM_001011954	1.93	291.6	10836788	cytochrome b reductase 1
Fdxr	NM_024153	1.29	41.7	10748909	ferredoxin reductase
**Epigenetics**					
RGD1564767	NM_001024282	1.24	67.7	10795196	Histone H2a
Adarb1	NM_012894	1.59	24.8	10829367	adenosine deaminase, RNA-specific, B1
Hdac8	NM_001126373	1.24	31.4	10938734	histone deacetylase 8
LOC308670	AB191343	1.68	357.7	10707548	pink-eyed dilution
Myst3	NM_001100570	0.81	-98.9	10792402	MYST histone acetyltransferase (monocytic leukemia) 3

**Golgi Apparatus**					
LOC685079	XM_001062200	1.29	58.2	10842117	similar to Protein SYS1 homolog
Man1a1	NM_001033656	0.81	-85.1	10833394	mannosidase, alpha, class 1A, member 1
Cog7	NM_001033889	1.20	20.7	10725427	component of oligomeric golgi complex 7
**Growth Factors**					
Bmp3	NM_017105	0.55	-52.2	10775573	bone morphogenetic protein 3
Creg1	NM_001105966	1.35	53.3	10765335	cellular repressor of E1A-stimulated genes 1
Cx3cl1	NM_134455	0.64	-41.9	10809269	chemokine (C-X3-C motif) ligand 1
Ecop	NM_001108630	1.25	24.4	10855846	EGFR-coamplified and overexpressed protein
Megf6	NM_022955	1.25	39.2	10874458	multiple EGF-like-domains 6
Rspo1	NM_001107980	0.82	-20.7	10871898	R-spondin homolog (Xenopus laevis)
**Immune Response**					
Cd55	NM_022269	0.72	-105.0	10767388	Cd55 molecule
Igsf9	NM_001107197	0.76	-32.5	10765751	immunoglobulin superfamily, member 9
Spag5	NM_001044224	1.31	32.1	10745107	sperm associated antigen 5
**Metabolism & Transport**					
Acadm	NM_016986	1.21	74.5	10827454	acyl-Coenzyme A dehydrogenase, C-4 to C-12 straight chain
Acsf2	NM_001034951	1.54	51.1	10746399	acyl-CoA synthetase family member 2
Agpat2	NM_001107821	1.45	80.4	10843838	1-acylglycerol-3-phosphate O-acyltransferase 2 (lysophosphatidic acid acyltransferase, beta)
Akr1cl2	NM_001008342	1.36	36.7	10796027	aldo-keto reductase family 1, member C-like 2
Aqp9	NM_022960	0.73	-13.1	10918569	aquaporin 9
Asl	NM_021577	1.22	42.2	10757962	argininosuccinate lyase
B3galt6	NM_001106699	1.32	15.1	10882246	UDP-Gal:betaGal beta 1,3-galactosyltransferase, polypeptide 6
B3gnt2	NM_001107240	1.25	207.8	10778708	UDP-GlcNAc:betaGal beta-1,3-N-acetylglucosaminyltransferase 2
Doc2b	NM_031142	1.24	36.5	10744970	double C2-like domains, beta
Elovl2	NM_001109118	1.65	59.7	10794609	elongation of very long chain fatty acids (FEN1
Exosc2	NM_001108952	1.31	46.1	10835396	exosome component 2
Esyt1	NM_017249	1.20	39.7	10899793	extended synaptotagmin-like protein 1
Farsa	NM_001024237	1.20	42.9	10810045	phenylalanyl-tRNA synthetase, alpha subunit
Gja1	NM_012567	0.83	-155.3	10830189	gap junction protein, alpha 1
Gsta4	NM_001106840	1.23	299.8	10884917	glutathione S-transferase alpha 4
Hbb	NM_033234	1.28	187.6	10724315	hemoglobin, beta
Heph	NM_133304	1.24	71.6	10934073	hephaestin
Hmgcs2	NM_173094	1.26	136.0	10817759	3-hydroxy-3-methylglutaryl-Coenzyme A synthase 2 (mitochondrial)
Hsd17b2	NM_024391	1.26	53.4	10811341	hydroxysteroid (17-beta) dehydrogenase 2
LOC501110	NM_001024361	1.23	211.7	10926967	similar to Glutathione S-transferase A1 (GTH1) (HA subunit 1) (GST-epsilon) (GSTA1-1) (GST class-alpha)
LOC680026	XM_001055503	1.33	24.8	10803205	similar to ATP synthase, H+ transporting, mitochondrial F0 complex, subunit G
MGC72973	NM_198776	1.28	164.8	10724319	beta-glo
Mocs3	NM_001107804	1.30	23.3	10842493	molybdenum cofactor synthesis 3
Mpst	NM_138843	1.33	31.3	10897446	mercaptopyruvate sulfurtransferase
Naaa	NM_001010967	1.20	11.6	10771690	N-acylethanolamine acid amidase
Nampt	NM_177928	1.41	90.7	10884162	nicotinamide phosphoribosyltransferase
Nat9	NM_001134835	1.23	33.6	10748882	N-acetyltransferase 9 (GCN5-related, putative)
Nosip	NM_001106260	1.26	33.2	10706712	nitric oxide synthase interacting protein
Nppc	NM_053750	1.44	29.3	10929606	natriuretic peptide precursor C
Pctp	NM_017225	1.29	15.3	10746201	phosphatidylcholine transfer protein
Pcyt1a	NM_078622	1.32	37.3	10754813	phosphate cytidylyltransferase 1, choline, alpha
Pisd	ENSRNOT00000024813	1.21	73.5	10773650	phosphatidylserine decarboxylase
Pitpnm3	XM_220629	0.82	-14.4	10744641	PITPNM family member 3
Ppapdc1a	XM_219376	0.76	-11.7	10711454	phosphatidic acid phosphatase type 2 domain containing 1A
Ppt1	NM_022502	1.29	125.5	10871695	palmitoyl-protein thioesterase 1
Ptgs1	NM_017043	1.50	16.6	10835817	prostaglandin-endoperoxide synthase 1

Pyroxd2	NM_001004261	1.32	43.1	10715400	pyridine nucleotide-disulphide oxidoreductase domain 2
Slc22a5	NM_019269	1.26	74.4	10742645	solute carrier family 22 (organic cation
Slc25a20	NM_053965	1.25	115.2	10913218	solute carrier family 25 (carnitine
Slc26a7	NM_001106638	1.46	77.2	10875581	solute carrier family 26, member 7
Slc27a3	NM_001106439	1.25	16.8	10824611	solute carrier family 27 (fatty acid transporter), member 3
Slc38a10	ENSRNOT00000006136	1.21	45.8	10749594	solute carrier family 38, member 10
Slc4a4	NM_053424	0.77	-139.6	10775997	solute carrier family 4 (anion exchanger), member 4
Thnsl2	NM_001009658	1.22	47.1	10863093	threonine synthase-like 2 (S. cerevisiae)
Timm22	AF223951	1.22	42.4	10745484	translocase of inner mitochondrial membrane 22 homolog (yeast)
Tmc1	NM_001108521	0.79	-15.8	10729293	transmembrane channel-like 1
Tmed1	NM_001013432	1.21	23.2	10915561	transmembrane emp24 protein transport domain containing 1
Tut1	NM_001033901	1.35	107.2	10713779	terminal uridylyl transferase 1, U6 snRNA-specific
Vamp5	NM_053555	1.26	10.6	10863215	vesicle-associated membrane protein 5
**Proteolysis**					
Capn8	NM_133309	1.31	96.9	10766400	calpain 8
Cpa4	NM_001109346	0.82	-13.2	10854303	carboxypeptidase A4
Dpp3	NM_053748	1.21	29.5	10727701	dipeptidylpeptidase 3
Klk7	NM_001106254	1.29	10.8	10706431	kallikrein-related peptidase 7
Mmp15	NM_001106168	0.73	-55.7	10809136	matrix metallopeptidase 15
Mmp3	NM_133523	0.68	-23.0	10907881	matrix metallopeptidase 3
Rnf213	XM_001081768	0.64	-276.6	10739984	ring finger protein 213
Serpina9	NM_001106754	0.81	-43.8	10891977	serine (or cysteine) peptidase inhibitor, clade A (alpha-1 antiproteinase, antitrypsin), member 9
Serpinb8	NM_001105948	1.83	15.4	10763385	serpin peptidase inhibitor, clade B (ovalbumin), member 8
Thop1	NM_172075	1.26	41.4	10900344	thimet oligopeptidase 1
Tysnd1	NM_001108932	1.28	26.5	10830058	trypsin domain containing 1
Vps33a	NM_022961	1.25	69.5	10758415	vacuolar protein sorting 33 homolog A (S. cerevisiae)
**Receptors & Binding Proteins**					
Aebp1	NM_001100970	1.87	102.4	10774015	AE binding protein 1
Axl	NM_031794	0.82	-100.6	10719900	Axl receptor tyrosine kinase
Fgfr3	NM_053429	1.55	40.9	10777748	fibroblast growth factor receptor 3
Lrp2	NM_030827	0.77	-45.9	10845977	low density lipoprotein-related protein 2
Ngfr	NM_012610	1.45	39.9	10746538	nerve growth factor receptor (TNFR superfamily, member 16)
Tacr3	NM_017053	1.89	27.8	10819139	tachykinin receptor 3
Unc5b	NM_022207	1.82	22.2	10833013	unc-5 homolog B (C. elegans)
Fzd1	NM_021266	1.21	33.8	10853469	frizzled homolog 1 (Drosophila)
Gfra3	NM_053398	1.24	12.5	10803815	GDNF family receptor alpha 3
Igfbp5	NM_012817	1.43	1484.5	10928837	insulin-like growth factor binding protein 5
Ldlrap1	NM_001109271	1.23	29.9	10880552	low density lipoprotein receptor adaptor protein 1
LOC683973	NM_001000108	0.81	-19.2	10809866	similar to spermatogenesis associated glutamate (E)-rich protein 4b
PVR	NM_017076	0.79	-17.5	10719616	poliovirus receptor
P2rx2	NM_053656	1.32	12.4	10759383	purinergic receptor P2X, ligand-gated ion channel, 2
Sorcs3	NM_001106367	2.74	94.7	10716026	sortilin-related VPS10 domain containing receptor 3
Tgfbr3	NM_017256	0.80	-70.9	10771070	transforming growth factor, beta receptor III
**Signaling**					
Adck5	NM_001135798	1.22	43.6	10897308	aarF domain containing kinase 5
Alpk3	ENSRNOT00000015898	1.32	15.4	10708247	alpha-kinase 3
Calhm2	NM_001008306	1.79	63.8	10730636	calcium homeostasis modulator 2
Calml3	NM_001012054	1.84	118.7	10796090	calmodulin-like 3
Cdc42ep1	NM_001079700	0.68	-33.1	10897480	CDC42 effector protein (Rho GTPase binding) 1
Dapp1	NM_001108568	0.78	-14.2	10826985	dual adaptor of phosphotyrosine and 3-phosphoinositides
Dock5	NM_001107274	1.28	24.0	10784751	dedicator of cytokinesis 5
Dok4	NM_001108438	1.86	124.9	10805976	docking protein 4
Egfl6	NM_001108254	2.23	261.2	10933349	EGF-like-domain, multiple 6
Fxyd3	NM_172317	2.18	32.6	10720884	FXYD domain-containing ion transport regulator 3

Grik1	NM_001111117	1.63	10.0	10752919	glutamate receptor, ionotropic, kainate 1
Grik5	NM_031508	0.78	-26.3	10719754	glutamate receptor, ionotropic, kainate 5
Ip6k1	NM_053316	1.21	75.2	10913134	inositol hexakisphosphate kinase 1
Itga6	ENSRNOT00000045394	0.80	-328.3	10836849	integrin, alpha 6
Itgb8	NM_001108726	0.70	-33.7	10892835	integrin, beta 8
Mink1	BC090005	0.82	-44.4	10735280	misshapen-like kinase 1 (zebrafish)
Obscn	XM_340807	0.79	-10.8	10743027	obscurin, cytoskeletal calmodulin and titin-interacting RhoGEF
Pbxip1	NM_001100976	1.24	29.3	10816807	pre-B-cell leukemia homeobox interacting protein 1
Pde11a	NM_001127481	0.77	-11.8	10846301	phosphodiesterase 11A
Pde1b	NM_022710	0.83	-77.1	10899676	phosphodiesterase 1B, calmodulin-dependent
Pfkp	NM_206847	1.56	70.5	10795989	phosphofructokinase, platelet
Phospho2	NM_001007642	1.21	38.1	10836633	phosphatase, orphan 2
Pih1d1	NM_001024868	1.23	25.3	10706724	PIH1 domain containing 1
Pik3ip1	NM_001017453	1.24	33.7	10773695	phosphoinositide-3-kinase interacting protein 1
Ppm1k	NM_001107863	1.28	161.7	10855853	protein phosphatase 1K (PP2C domain containing)
Rabgap1l	NM_001107190	1.23	87.4	10769138	RAB GTPase activating protein 1-like
Rap2b	NM_133410	1.22	22.3	10815659	RAP2B, member of RAS oncogene family
Rarg	NM_001135249	1.28	18.0	10907574	retinoic acid receptor, gamma
Rassf4	NM_001024275	1.83	48.4	10864874	Ras association (RalGDS
Rics	ENSRNOT00000011589	0.81	-86.5	10908863	Rho GTPase-activating protein
Scube1	NM_001134884	1.44	77.8	10905843	signal peptide, CUB domain, EGF-like 1
Smad6	NM_001109002	2.83	244.3	10918186	SMAD family member 6
Smad7	NM_030858	1.74	34.2	10802734	SMAD family member 7
Smad9	NM_138872	2.17	94.2	10815436	SMAD family member 9
Spry3	NM_001109063	0.75	-10.7	10760805	sprouty homolog 3 (Drosophila)
Srgap3	ENSRNOT00000009066	0.82	-65.6	10864590	SLIT-ROBO Rho GTPase activating protein 3
Tek	NM_001105737	1.21	26.9	10869946	TEK tyrosine kinase, endothelial
Tom1	NM_001008365	1.21	24.5	10806106	target of myb1 homolog (chicken)
**DNA Repair**					
Bcr	ENSRNOT00000001766	0.75	-35.0	10832602	breakpoint cluster region
Smug1	NM_177934	1.25	27.8	10907659	single-strand-selective monofunctional uracil-DNA glycosylase 1
**Transcription**					
Brpf3	NM_001107615	0.82	-25.7	10828791	bromodomain and PHD finger containing, 3
Creb3l1	NM_001005562	0.73	-16.6	10847474	cAMP responsive element binding protein 3-like 1
Crtc3	ENSRNOT00000015948	0.80	-25.3	10723196	CREB regulated transcription coactivator 3
Ddx60	XP_001070139	0.82	-190.1	10791358	DEAD (Asp-Glu-Ala-Asp) box polypeptide 60
Fat1	NM_031819	0.83	-80.0	10791786	FAT tumor suppressor homolog 1 (Drosophila)
Id1	NM_012797	1.25	115.7	10840890	inhibitor of DNA binding 1
Id3	NM_013058	1.45	263.7	10872972	inhibitor of DNA binding 3
LOC682968	ENSRNOT00000061079	1.21	14.6	10806085	similar to Retinal homeobox protein Rx (DRx1) (DRx)
Med4	NM_001024256	1.40	113.7	10781443	mediator complex subunit 4
Msx2	NM_012982	1.86	40.0	10794200	msh homeobox 2
Ndrg2	NM_133583	1.24	221.1	10783213	N-myc downstream regulated gene 2
Nfatc2ip	NM_001007692	1.22	37.3	10725675	nuclear factor of activated T-cells, cytoplasmic, calcineurin-dependent 2 interacting protein
Nfix	NM_030866	0.77	-84.5	10806665	nuclear factor I
Nfkbiz	NM_001107095	0.78	-18.4	10750848	nuclear factor of kappa light polypeptide gene enhancer in B-cells inhibitor, zeta
Nr1d1	NM_001113422	0.74	-18.9	10746955	nuclear receptor subfamily 1, group D, member 1
Nudt6 ///Fgf2	NM_181363 /// M22427	1.26	17.6	10822929	nudix (nucleoside diphosphate linked moiety X)-type motif 6 /// fibroblast growth factor 2
Poll	NM_001014168	1.20	16.9	10730419	polymerase (DNA directed), lambda
Ralb	NM_053821	1.42	158.0	10767098	v-ral simian leukemia viral oncogene homolog B (ras related; GTP binding protein)
RGD1560095	ENSRNOT00000040584	0.60	-51.2	10833564	similar to double homeobox, 4
Supt3h	ENSRNOT00000027293	1.32	32.8	10926627	suppressor of Ty 3 homolog (S. cerevisiae)

Tet3	ENSRNOT00000031312	0.81	-22.9	**10863523**	tet oncogene family member 3
Tiam1	NM_001100558	0.75	-17.0	10750144	T-cell lymphoma invasion and metastasis 1
Ttf2	NM_001106454	1.21	28.7	10825418	transcription termination factor, RNA polymerase II
Zfhx3	ENSRNOT00000019408	0.80	-24.0	10807688	zinc finger homeobox 3
Zim1	NM_001107473	0.76	-16.5	10703850	zinc finger, imprinted 1
Znf286a	ENSRNOT00000004294	1.28	18.9	10743608	zinc finger protein 286A
Znf692	NM_001126272	1.45	74.7	10733812	zinc finger protein 692
**Translation & Protein Modification**					
Lmf1	ENSRNOT00000000246	1.22	39.1	10732427	lipase maturation factor 1
mrpl9	NM_001007696	1.26	122.2	10817252	mitochondrial ribosomal protein L9
Oasl	NM_001009681	0.68	-36.2	10762740	2'-5'-oligoadenylate synthetase-like
Padi3	NM_017230	1.56	12.7	10880977	peptidyl arginine deiminase, type III
Pcbp4	ENSRNOT00000017139	1.29	60.6	10912849	poly(rC) binding protein 4
RGD1559877	ENSRNOT00000049689	1.25	10.7	10800511	similar to 60S ribosomal protein L29 (P23)
RGD1560949	XM_001078650	1.28	12.5	10808338	similar to testis nuclear RNA-binding protein-like
Rnaseh2a	NM_001013234	1.22	11.7	10806587	ribonuclease H2, subunit A
Sf1	NM_001110793	0.79	-104.6	10713362	splicing factor 1
Sfrs12ip1	NM_001008373	1.23	51.3	10812865	SFRS12-interacting protein 1
Sh3bp5l	NM_001127581	1.22	24.6	10733829	SH3 binding domain protein 5 like
Tram1l1	NM_001107724	1.27	12.9	10818892	translocation associated membrane protein 1-like 1
Tsen54	NM_001109576	1.22	17.2	10739630	tRNA splicing endonuclease 54 homolog (S. cerevisiae)
**Miscellaneous & Unknown**					
Dcbld1	ENSRNOT00000000462	1.20	37.3	10830135	discoidin, CUB and LCCL domain containing 1
Fam123b	NM_001109320	0.80	-47.0	10938485	family with sequence similarity 123B
Fam132a	NM_001108000	1.87	685.6	10874811	family with sequence similarity 132, member A
Fam20c	NM_001012238	1.26	18.4	10760738	family with sequence similarity 20, member C
Fam82b	NM_001031663	1.33	23.6	10867799	family with sequence similarity 82, member B
Gatsl3	NM_001025128	1.27	18.3	10773838	GATS protein-like 3
Giyd2	NM_001009292	1.20	15.2	10725795	GIY-YIG domain containing 2
LOC681219	ENSRNOT00000038757	1.23	119.7	10899197	hypothetical protein LOC681219
RGD1564317	XM_579774	0.81	-40.9	10752970	RGD1564317
LOC685424	ENSRNOT00000050888	0.83	-94.6	10806494	hypothetical protein LOC685424
LOC501482	ENSRNOT00000047230	0.83	-82.5	10806198	hypothetical gene supported by BC082068
Lrrn4	XM_230550	0.72	-135.0	10850170	leucine rich repeat neuronal 4
Lin37	NM_001106245	1.20	57.9	10720648	lin-37 homolog (C. elegans)
Morn2	NM_001126086	1.21	11.2	10882511	MORN repeat containing 2
Morn4	NM_001024975	1.24	36.5	10715455	MORN repeat containing 4
Rftn2	ENSRNOT00000020910	1.24	25.9	10928167	raftlin family member 2
RGD1566386	NM_001107127	1.38	41.5	10760749	similar to Hypothetical protein A430033K04
RGD1307722	NM_001108730	1.51	326.1	10900201	similar to hypothetical protein MGC20700
RGD1310271	XM_233727	2.18	72.1	10882356	similar to hypothetical protein MGC45873
RGD1310778	NM_001127523	1.22	21.1	10753017	similar to Putative protein C21orf45
RGD1565890	ENSRNOT00000003157	1.30	100.8	10740396	similar to RUN domain containing 2A
Spryd4	NM_001037765	1.26	28.5	10899736	SPRY domain containing 4
Ttc30b	NM_001127607	1.22	21.7	10846293	tetratricopeptide repeat domain 30B
Ttc38	NM_001130499	1.25	26.7	10898368	tetratricopeptide repeat domain 38
Tmco6	NM_001106154	1.35	60.5	10801074	transmembrane and coiled-coil domains 6
Wipi1	NM_001127297	1.50	96.6	10739313	WD repeat domain, phosphoinositide interacting 1
Wwc2	NM_001109111	1.66	171.6	10788101	WW and C2 domain containing 2
**EST's**					
RGD1306839	NM_001106347	1.28	13.9	10729604	similar to RIKEN cDNA 5033414D02
RGD1306962	ENSRNOT00000021944	1.21	45.6	10717311	similar to dJ55C23.6 gene product
RGD1311186	NM_001106310	1.20	11.7	10726604	similar to RIKEN cDNA 1810014F10 gene
RGD1566239	NM_001107305	1.22	10.3	10787549	similar to RIKEN cDNA 2810428I15
	---	2.24	48.0	10788037	

	---	1.52	26.9	10883880	
	---	1.41	23.8	10870979	
	---	1.40	16.8	10859796	
	---	1.38	427.3	10820324	
	---	1.37	21.5	10867662	
	---	1.36	21.1	10752061	
	---	1.36	27.4	10718413	
	---	1.30	44.3	10902320	
	---	1.30	42.0	10869202	
	---	1.28	34.5	10833366	
	---	1.26	10.4	10938981	
	---	1.25	19.5	10806777	
	---	1.25	26.4	10718433	
	---	1.24	14.6	10871464	
	---	1.23	36.5	10814892	
	---	1.22	11.2	10768134	
	---	0.82	-13.4	10936432	
	---	0.82	-15.1	10817126	
	---	0.82	-74.9	10824788	
	---	0.81	-27.7	10708649	
	---	0.80	-54.0	10790465	
	---	0.79	-11.4	10934770	
	---	0.79	-14.4	10712863	
	---	0.79	-145.6	10851813	
	---	0.78	-11.0	10922843	
	---	0.78	-121.7	10796230	
	---	0.78	-205.3	10719074	
	---	0.78	-205.3	10746652	
	---	0.77	-70.6	10726672	
	---	0.77	-22.1	10707705	
	---	0.77	-11.0	10919373	
	---	0.76	-20.7	10936458	
	---	0.75	-81.5	10933753	
	---	0.71	-20.4	10914903	
	---	0.66	-134.8	10767985	
	---	0.62	-60.6	10788002	
