# Supplementary material for: Inhibitory Actions of Anti-Müllerian Hormone (AMH) on Ovarian Primordial Follicle Assembly
Source: PLoS One. 2011 May 27;6(5):e20087. doi: 10.1371/journal.pone.0020087 (PMC3103528; doi:10.1371/journal.pone.0020087)
Supplement: Table S2 — Top cell processes for 274 differentially expressed genes in AMH-treated P0-ovary. Cell processes with highest local connectivity values (number of related literature references) were extracted from shortest connection subnetwork obtained with Pathway Studio 7.0 software (Ariadne Genomics, Inc., Rockville, MD). (PDF) [file pone.0020087.s005.pdf]

Supplemental Table S2.

**Gene Category**	**Local Connectivity**
Cell proliferation	70
Cell differentiation	62
Apoptosis	57
Cell cycle	35
Cell survival	34
Cell death	33
Endocytosis	24
Cell migration	22
Morphogenesis	21
Angiogenesis	20
Wound healing	18
Neurite outgrowth	17
Cell contact	16
Cell fate	16
S phase	15
Mitosis	15
Embryonic development	14
DNA recombination	13
Senescence	13
Cell cell contact	12
Pregnancy	12
Membrane polarization	12
Meiosis	12
DNA degradation	11
Blood pressure	11
Neurogenesis	11
Exocytosis	10
Synaptic transmission	10

Table S2. Top cell processes for 274 differentially expressed genes in AMH-treated P0-ovary. Cell processes with highest local connectivity values (number of related literature references) were extracted from shortest connection sub-network obtained with Pathway Studio 7.0 software (Ariadne Genomics, Inc., Rockville, MD)
